# Membrane nanodomains to shape plant cellular functions and signaling

**DOI:** 10.1111/nph.20367

**Published:** 2024-12-25

**Authors:** Omar Hdedeh, Caroline Mercier, Arthur Poitout, Alexandre Martinière, Enric Zelazny

**Affiliations:** ^1^ IPSiM, Univ Montpellier, CNRS, INRAE, Institut Agro Montpellier 34000 France

**Keywords:** lipids, nanodomain, nanoenvironment, plant, plasma membrane, protein–protein interactions, signaling

## Abstract

Plasma membrane (PM) nanodomains have emerged as pivotal elements in the regulation of plant cellular functions and signal transduction. These nanoscale membrane regions, enriched in specific lipids and proteins, behave as regulatory/signaling hubs spatially and temporally coordinating critical cellular functions. In this review, we first examine the mechanisms underlying the formation and maintenance of PM nanodomains in plant cells, highlighting the roles of PM lipid composition, protein oligomerization and interactions with cytoskeletal and cell wall components. Then, we discuss how nanodomains act as organizing centers by mediating protein–protein interactions that orchestrate essential processes such as symbiosis, defense against pathogens, ion transport or hormonal and reactive oxygen species (ROS) signaling. Finally, we introduce the concept of nanoenvironments, where localized physicochemical variations are generated in the very close proximity of PM nanodomains, in response to stimuli. After decoding by a dedicated machinery likely localized in the vicinity of nanodomains, this enrichment of secondary messengers, such as ROS or Ca^2+^, would allow specific downstream cellular responses. This review provides insights into the dynamic nature of nanodomains and proposes future research to better understand their contribution to the intricate signaling networks that govern plant development and stress responses.

**Table:** 

	Contents	
	Summary	1369
I.	Introduction	1370
II.	Membrane and nanodomain emerging properties	1370
III.	Nanodomains: pre‐existing structures and/or auto‐assembly?	1371
IV.	Lipids are key regulators of PM organization into nanodomains	1371
V.	Role of protein oligomerization in nanodomain formation	1373
VI.	Cortical cytoskeleton meshwork and nanodomains	1373
VII.	Cell wall/PM interactions act as an additive layer of regulation for nanodomain organization	1374
VIII.	Functional link between PM nanodomain organization and endocytosis	1374
IX.	Organization of proteins in nanodomains to coordinate/modulate their functions	1375
X.	From nanodomains to nanoenvironments	1378
XI.	Conclusion and future prospective	1380
	Acknowledgements	1381
	References	1381

## I. Introduction

In the crowded environment of the cell, where for instance protein concentration can reach several hundred mg ml^−1^ in the cytoplasm, highly complex biochemical reactions are constantly ongoing (Zimmerman & Trach, 1991; Liu *et al*., 2022). To be efficient, timely and spatially defined, biochemical reactions need to be compartmentalized. This phenomenon is best exemplified in the case of membranes that define cells and organelles. Indeed, their surfaces participate in biochemical reaction networks by confining proteins or lipids, aligning reaction partners and directly controlling enzymatic activities. At the cellular level, membrane‐localized reactions shape cellular membranes and could generate signaling gradients that originate from the plasma membrane (PM) and extend into the cytoplasm and the nucleus. Therefore, membranes appear as essential platforms from which many cellular processes are scaffolded, leading ultimately to developmental and physiological responses of organisms.

At the molecular scale, the thermic agitation should lead to an even concentration of the membrane constituents, meaning that at any place of the membrane, the quantity of biomolecules should be the same. However, after decades of studies, biological membranes appear instead to be highly heterogeneous both spatially and temporally. Membranes are now considered as a juxtaposition of domains of small size composed of different lipids and proteins. Throughout this review, we will refer to them by the term nanodomain as a local assembly of proteins and/or lipids that display a nanoscale size (< 1 μm in diameter) (Jaillais *et al*., 2024). Nanodomains are highly diverse in their composition and are present in virtually all the types of membranes including the PM, the endoplasmic reticulum and chloroplast thylakoid membranes (Johnson *et al*., 2014; Gao *et al*., 2019; Smokvarska *et al*., 2020). Nanodomains were shown to be involved in the regulation of many cellular processes by acting as regulatory/signaling platforms (Demir *et al*., 2013; Gronnier *et al*., 2017; Platre *et al*., 2019). Contrary to what was sometimes proposed in the past, nanodomains do not imply predefined physicochemical properties like being enriched in sphingolipids, sterols or phosphoinositides, even if these lipids can be components, among others, of some nanodomains. Similarly, nanodomains are not obligatory associated with specific lipid order nor detergent resistant membrane (DRM) fraction (Mongrand *et al*., 2010; Tanner *et al*., 2011).

In recent years, plant PM nanodomains have emerged as key elements to regulate a variety of biological functions by shaping molecular reactions and acting on cell signaling. In this review, we will first detail the genesis and maintenance of plant PM nanodomains. Second, we will illustrate their biological roles as regulatory/signaling hubs gathering functionally related proteins. Finally, we will emphasize the ability of PM nanodomains to create in their very close proximity localized variations of physicochemical parameters, hereafter called nanoenvironments that may act on signal transduction processes.

## II. Membrane and nanodomain emerging properties

**Fig. 1:**
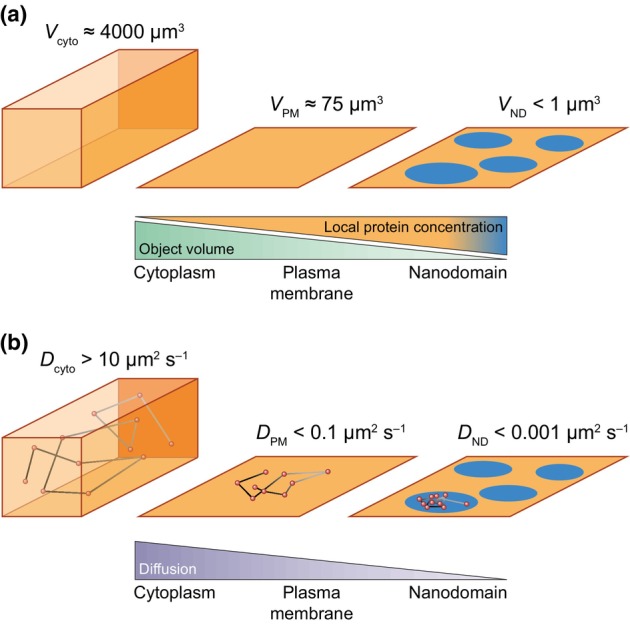
Confinement in nanodomains influences biochemical reactions by increasing the local concentration of biomolecules, such as proteins, as well as reducing their diffusion. (a) Confining proteins in a thin layer at the membrane surface or within membrane nanodomains increases their concentration. (b) Association of proteins with membranes and even more so within nanodomains drastically reduces their diffusion. *V* and *D* correspond to volume and diffusion, respectively. cyto, cytoplasm; ND, nanodomain; PM, plasma membrane.

Biomolecules binding to membranes have a drastic effect on their kinetic of biochemical interactions (Küchler *et al*., 2016; Leonard *et al*., 2023). First, membrane binding is changing biomolecule local concentration. For instance, for an Arabidopsis differentiated root cell displaying a cytoplasmic volume of *c*. 4000 μm^3^ (Dünser *et al*., 2019), the corresponding PM inner leaflet only represents 75 μm^3^ (with 5 nm PM thickness). This is increasing the local reactant concentration to 50 times (Fig. 1a). The exact same reasoning can be followed for biomolecules clustering in nanodomains where the volume will be even smaller (Fig. 1a). Membrane binding also reduces the dimensionality of molecules. Indeed, when a protein is bound to the membrane, its rotation is restricted to the axis perpendicular to the membrane surface, which enhances biochemical reactions by aligning reaction partners (Kholodenko *et al*., 2000). Thus, confining proteins in membranes or within nanodomains will modify biomolecules apparent affinity, enabling protein interactions that would be impossible in solution (McCloskey & Poo, 1986). Second, membrane binding can act on diffusion, which is typically decreased by 100–1000 times between the cytoplasm and membranes (Fig. 1b; McCloskey & Poo, 1986). In addition, biomolecule clusterization in nanodomains could decrease their diffusion even more. In extreme cases, reactants could be virtually immobilized and their reaction rate will then tend to zero (Fig. 1b). Membrane association and nanodomain organization is also acting on rotational motion of biomolecules and could help to co‐align interactants (Kholodenko *et al*., 2000). In the case of reactants that are reversibly organized in nanodomains, interaction kinetics will depend on the time they spend in nanodomains. Because nanodomain organization may increase local concentration and decrease diffusion rate, fast nanodomain association rate will favor protein–protein interactions. Conversely, the rate of nanodomain association can become limiting for slow binding reactions. Therefore organization of biomolecules in nanodomains have a drastic effect on interaction kinetics and consequently on signaling pathways (Küchler *et al*., 2016; Mishra & Johnson, 2021).

## III. Nanodomains: pre‐existing structures and/or auto‐assembly?

As proposed by Jaillais *et al*. (2024), nanodomain constituents can be divided in two classes: ‘driver’ molecules that are necessary to form or maintain nanodomains and ‘client’ molecules that clusterize in nanodomains but could be dispensable for their maintenance. This concept is illustrated by the following examples. In response to auxin, the small GTPase Rho of Plant6 (ROP6) organizes in nanodomains that are enriched in the anionic lipid phosphatidylserine (PS) that helps ROP6 stabilization in nanodomains through electrostatic interaction with its C‐terminal lysine and arginine amino acids. Importantly, before any auxin stimulation, nanodomains of PS pre‐exist in the membrane. Thus, PS and ROP6 act as ‘driver’ and ‘client’ molecules, respectively (Platre *et al*., 2019). Some proteins named Remorins (REM) organize in PM nanodomains (Jarsch *et al*., 2014) and were also demonstrated to act as ‘driver’ molecules. Indeed, in *Medicago truncatula*, Symbiotic Remorin 1 (SYMREM1) is necessary to induce the organization of the receptor Lysine Motif Kianse3 (LYK3) into nanodomains, following rhizobial inoculation (Liang *et al*., 2018). This mechanism will be detailed hereafter.

Alternatively, to pre‐existing nanodomains, some nanodomains are *de novo* constituted in membranes. In this case, nanodomains are formed/maintained by a series of feedback loops creating reaction–diffusion processes (Schweisguth & Corson, 2019; Landge *et al*., 2020). In tip growing cells, like pollen tubes and root hairs, polar domains are proposed to be formed by self‐assembly (Klahre & Kost, 2006; Hwang *et al*., 2008, 2010; Luo *et al*., 2017). Self‐assembly seems also to drive the formation of nanodomains. Phosphatidylinositol 4‐phosphate 5‐kinases (PIP5Ks) physically interact with the Rho‐GTPase *Nicotiana tabacum* Ras‐related C3 botulinum toxin substrate 5 (NtRAC5) and create local phosphatidylinositol‐4,5‐bisphosphate (PI(4,5)P2) rich nanodomains in the pollen tube. In turn, this local enrichment in PI(4,5)P2 may favor NtRAC5 recruitment and stabilize actin through the activation of RAC effector proteins like ROP‐interactive CRIB motif‐containing protein (RIC) (Wu *et al*., 2001; Fratini *et al*., 2021; Heilmann & Heilmann, 2022). Armadillo Repeat Only (ARO) proteins form nanodomains and participate in the stabilization of growth sites in root hair cells (Kulich *et al*., 2020). ARO interacts with anionic lipids, ROP1 and ROP1 Enhancer (RENGAP). Because RENGAP proteins are negative regulators of ROPs, they are thought to act as negative feedback loops on ROP signaling in nanodomains (Kulich *et al*., 2020). Nagashima *et al*. (2018) proposed that feedback interactions between ROP, their activators guanidine exchange factors (GEFs) and their inhibitors GTPase activating proteins (GAPs), was sufficient to create periodic organization in the PM (Nagashima *et al*., 2018). More recently, the role of reaction–diffusion processes in ROP nanodomain formation was nicely extended to six different Arabidopsis ROPs (Sternberg *et al*., 2021; Deinum & Jacobs, 2024). In conclusion, the formation and maintenance of membrane nanodomains could arise from both self‐assembly and pre‐existing structures in plant PM.

## IV. Lipids are key regulators of PM organization into nanodomains

**Fig. 2:**
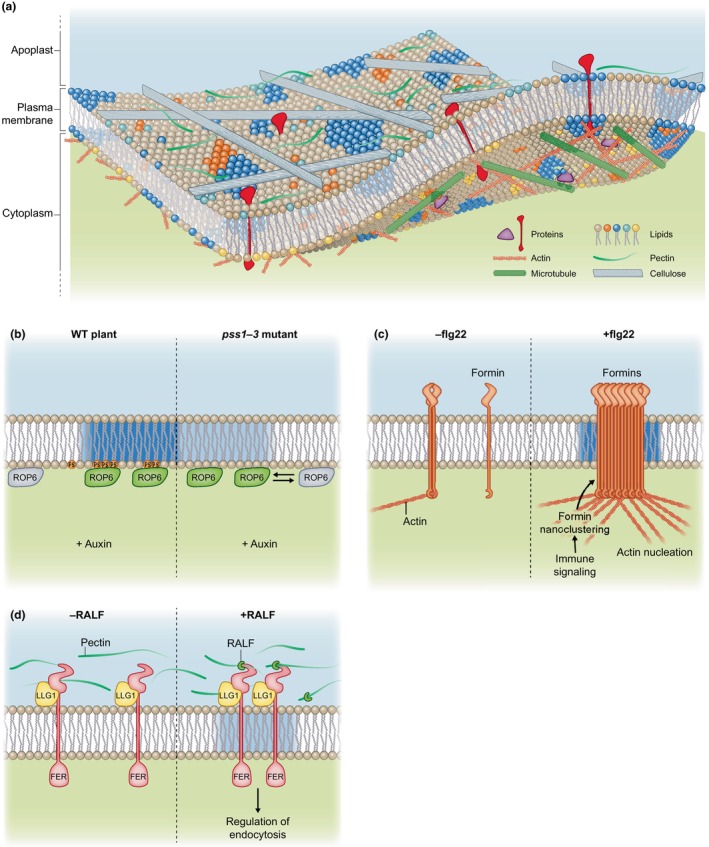
Mechanisms insuring the formation and the maintenance of plasma membrane (PM) nanodomains in plant cells. (a) Schematic representation of plant cell PM heterogeneity. The PM is highly heterogeneous and made up of a wide range of lipids and proteins. These elements can arrange into nanodomains of varying complexity. The cell wall, mostly composed of cellulose and pectin, has an important effect on protein organization in nanodomains. On the cytoplasmic side, the cytoskeleton, made up mainly of microtubules and actin microfilaments, also contributes to the stability of nanodomains. (b) Upon auxin stimulation of Arabidopsis root cells, Rho of Plant6 (ROP6) organizes in PM nanodomains. These domains contain phosphatidylserine (PS) that helps ROP6 stabilization through electrostatic interactions. In the absence of PS, as in the pss1‐3 mutant, ROP6 nanodomains are still present, but ROP6 stability in the domains is decreased (as represented by an inverted double arrow). (c) In response to flagellin, actin polymerization proteins named type‐I formin oligomerize and arrange in PM nanoclusters. This local condensation and stabilization activate formins that in turn induce actin nucleation. (d) The receptor‐like kinase FERONIA (FER) interacts with both pectins and rapid alkalinization factor (RALF) and acts as a cell growth regulator. Interestingly, upon abiotic stimulation, RALF and pectin phase separate and recruit FER together with the co‐receptor Lorelei‐Like GPI‐anchored protein 1 (LLG1) into PM nanodomains. This complex stimulates global endocytosis ensuring plant resilience under stress. WT, wild‐type.

In this section, we will describe the different cellular structures and mechanisms that are involved in the formation/maintenance of PM nanodomains (Fig. 2a). Several hundred of lipid species co‐exist in plant PM (Bahammou *et al*., 2024). Understanding how they act on membrane organization and therefore participate in signaling processes is a major challenge. In the past, the characterization of PM lipid domains relied mostly on the use of DRM fractionation and localization of chemically stained lipids in liposomes. So far, *in vivo* analyses of lipid organization in nanodomains remain scarce, especially in the plant field. However, approaches using inhibitors, biosensors and mutants already suggested that some membrane heterogeneities are maintained by a complex lipid homeostasis as exemplified by PS and ROP6 nanodomains, as explained previously (Fig. 2b). *In vitro* studies and molecular modeling showed that certain lipid interactions, for instance between sterols and sphingolipids, can create a liquid‐ordered phase (Gronnier *et al*., 2018; Mamode Cassim *et al*., 2019; Jaillais & Ott, 2020). Di‐4‐ANEPPDHQ (ANEPP) and other solvatochromic probes emerged as an interesting way to characterize membrane order phase *in vivo* (Gronnier *et al*., 2017; Grosjean *et al*., 2018; Huang *et al*., 2019; Pan *et al*., 2020). ANEPP is a ratiometric fluorescent probe that displays different emission p in liquid‐ordered phase and liquid‐disordered phase (non‐nanodomain). The use of ANEPP revealed a decrease of lipid order in the PM after methyl‐β‐cyclodextrin treatment, a sterol‐depleting agent or in the sterol biosynthesis mutant *fackel‐J79* (Pan *et al*., 2020). This suggests that lipid homeostasis is acting on membrane ordering and therefore potentially on membrane nanodomain formation/maintenance in plants. In the future, it will be interesting to extend those observations at the nanodomain scale as it was done in the animal field (Klymchenko, 2017; Pelletier *et al*., 2023). The role of sterols in the formation of PM nanodomains was also highlighted by other works showing for instance that the nanoclustering of *Solanum tuberosum* StREM1;3 is altered in the presence of the sterol synthesis inhibitor fenpropimorph (Gronnier *et al*., 2017). In a similar manner, the nanodomain organization of the auxin‐related Transmembrane Receptor Kinase 1 (TMK1) is dependent on sterols (Pan *et al*., 2020). Even if they label lipids in an indirect way, the emergence of specific lipid biosensors allows studying lipid localization in living cells. Among others, sensors are now available in plant cells for PS, PI(4,5)P2, phytosterol, and were all shown to form nanometric clusters in the PM (Furt *et al*., 2010; Platre *et al*., 2019; Fratini *et al*., 2021; Ukawa *et al*., 2022). Interestingly, certain lipids can probably be locally produced in plant PM by dedicated protein complexes anchored in nanodomains, for example PI4Kα or PI(4,5)P‐kinases (Fratini *et al*., 2021; Noack *et al*., 2022). In the future, lipid biosensors will provide an interesting way to study the importance of lipids in PM nanodomain organization. Performing ANEPP staining on giant unilamellar vesicles (GUVs) prepared with a mixture of the different classes of lipids found in the PM of BY‐2 cells, Grosjean *et al*. (2018) showed that the lipid composition influenced the GUV membrane order. In addition, the same GUVs containing only lipids displayed a different membrane order compared with giant vesicles of native PM, containing lipids and proteins, suggesting that proteins had an impact on membrane order. Those observations highlight the tight interplay between lipids and proteins in membranes and how it influences membrane properties.

## V. Role of protein oligomerization in nanodomain formation

In addition to PM lipid composition, protein oligomerization recently appeared as an important feature for nanodomain organization. REMs and members of the Stomatin/Prohibitin/Flotillin/HflK/C (SPFH) domain‐containing protein family arrange in nanodomains and were proposed to act as scaffolding proteins (Browman *et al*., 2007; Lefebvre *et al*., 2010; Martinière & Zelazny, 2021). Indeed, these two families of proteins form large oligomers that likely participate in the formation/maintenance of certain nanodomains (Daněk *et al*., 2016; Legrand *et al*., 2023). StREM1.3 trimer formation through their coiled‐coiled domain is needed for PM association (Martinez *et al*., 2019). AtREM1.3 multimerization is also maintained by the intrinsically disordered N‐terminal domain (Marín *et al*., 2012). Genetic, structural biology and modeling approaches showed that the C‐terminal anchor (REM‐CA) of StREM1.3 binds to sterols and phosphatidylinositol phosphate (PIP) insuring StREM1.3 nanodomains formation (Perraki *et al*., 2012; Gronnier *et al*., 2017; Legrand *et al*., 2019). From those interesting observations, it was tempting to speculate that StREM1.3 nanodomain formation may change PIP and/or sterol nanoclustering in plant cells. In accordance with this idea, REM overexpression increases membrane ordering and *rem1.2 × rem1.3* mutant displays an attenuated SA‐induced membrane ordering, as demonstrated using ANEPP (Huang *et al*., 2019). Another proof of the role of REM in nanodomain organization came from *in vitro* experiments showing that StREM1.3 drives lipid reorganization in reconstituted membranes, leading to the formation of REM‐enriched nanodomains, a process that is influenced by the phosphorylation of StREM1.3 intrinsically disordered domain by the Calcium‐dependent Protein Kinase 3 (CPK3) (Jolivet *et al*., 2023; Legrand *et al*., 2023). Although not directly related to nanodomain organization, REM oligomerization was recently shown to induce membrane blebs in protoplasts, suggesting a role of REM oligomerization in membrane topology (Su *et al*., 2023).

So far, the role of SPFH domain‐containing proteins in the organization of certain proteins or lipids in nanodomains was not formally demonstrated. However, as detailed hereafter, Flotillin 4 (FLOT4) was proposed to act as a central hub to organize symbiotic signaling in *M. truncatula* PM nanodomains (Liang *et al*., 2018). Flotillins are known to oligomerize via their C‐terminal domains (Yu *et al*., 2017) and Hypersensitive Induced Reaction (HIR) proteins, a plant specific SPFH protein family, are able to form large homo‐ and hetero‐oligomers *in vivo*, although the molecular function of such oligomerization process remains to be determined (Qi *et al*., 2011). Interestingly, in bacteria, the SPFH domain‐containing proteins HflK and HflC form a gigantic circular 24‐mer complex, featuring a laterally segregated membrane nanodomain (20 nm in diameter) bordered by transmembrane domains of HflK/C (C. Ma *et al*., 2022). This study provides new exciting insights in the contribution of SPFH domain‐containing proteins in the formation of membrane nanodomains.

## VI. Cortical cytoskeleton meshwork and nanodomains

Plasma membrane organization not only emerges from its lipid and protein composition but also from its interaction with the surrounding structures. This idea was theorized by the group of Kusumi (Tomishige *et al*., 1998; Ritchie *et al*., 2003; Kusumi *et al*., 2005). In the picket and fence model, protein and lipid diffusion in the PM is strongly influenced by the cytoskeleton, which is in direct contact with the PM, thanks to a putative binding between cytoskeleton and membrane‐anchored proteins. According to this model, actin filaments act as a barrier to diffusion of lipids and proteins from the inner face of the PM. These fences limit diffusion and therefore could participate in the creation of domains. In addition, the putative transmembrane proteins that hold actin microfilaments at the PM act as pickets and because they extend over the two lipid layers would also limit the movement of lipids even in the outer membrane leaflet. Interestingly, in this model, protein and lipid diffusion is constrained not because of specific interaction with the membrane skeleton, but thanks to the steric hindrance generated by the pickets and fences.

Compared with animal models, the steric hindrance effect of the cytoskeleton in plant cells seems to be limited, as the diffusion of artificial PM protein is not affected by cytoskeleton depolymerization drugs (Lenne *et al*., 2006; Martinière *et al*., 2012). Moreover, certain proteins known to be organized in nanodomains, for example FLOT2, HIR1 and PIN‐formed 3 (PIN3), are not affected by a disruption of the cytoskeleton (McKenna *et al*., 2019; Daněk *et al*., 2020). However, in other cases, like with the auxin efflux transporter PIN2 that forms nanodomains in the apico‐basal root cell membrane, 3D confocal imaging and immuno‐gold labeling show that PIN2 nanoclustering is sensitive to pharmacological and genetic removal of microtubules (Kleine‐Vehn *et al*., 2011; Li *et al*., 2021). Destabilization of actin also induces a reduction in SYMREM1 nanodomain density in the PM (Liang *et al*., 2018). In addition, the actin and microtubule cytoskeleton also acts on the diffusion of nanodomains containing the pathogen receptor Flagellin‐Sensing 2 (FLS2) (McKenna *et al*., 2019). In conclusion, the effect of the cytoskeleton as a diffusion barrier in plant cells seems to depend on the proteins and we think that an in‐depth analysis is still required to precisely decipher these mechanisms.

Some examples in the literature show specific interactions between the cytoskeleton and given biomolecules, which consequently control nanodomain organization. Cellulose Synthase (CesA) complexes are responsible for cellulose microfibril secretion and form nanodomains in the PM (Desprez *et al*., 2007; Crowell *et al*., 2009). Their function is tightly associated with microtubules and actin. Cellulose Synthase Interacting 1/POM2 and Korrigan (KOR) form bridges between CesA complexes and microtubules, participating in CesA nanodomain maintenance and/or dynamics in the PM (Paredez *et al*., 2008; Bringmann *et al*., 2012; Vain *et al*., 2014). During immune signaling, type‐I formin, that contains single pass transmembrane domain and an intrinsic actin bundling activity, undergoes nanodomain formation connecting actin cytoskeleton regulation and membrane organization (Fig. 2c; Ma *et al*., 2021; Z. Ma *et al*., 2022). Similarly, PIP5K nanodomains in pollen tube regulate actin dynamics and organization by recruitment of RAC5 (Fratini *et al*., 2021). This shows that RAC5 or formin nanodomain organization can shape actin microfilaments. Networked 3C (NET3C), Kinesin Light Chain‐Related protein‐1 (KLCR1) and IQ‐Domain 2 (IQD2) together with Vesicle‐Associated Protein 27 (VAP27) arrange in nanodomains at ER/PM contact sites (Wang *et al*., 2014; Zang *et al*., 2021). This complex forms oligomers and are associated with actin microfilaments and microtubules. Interestingly, in this case, the cytoskeleton seems to be important for NET3C and VAP27 turn‐over within nanodomains, suggesting a role for the cytoskeleton to immobilized those proteins in nanodomains (Wang *et al*., 2014). We can hypothesize that the membrane nucleation machinery of the cytoskeleton may both polymerize actin or tubulin and simultaneously act as a priming event to initiate the formation of nanodomains.

## VII. Cell wall/PM interactions act as an additive layer of regulation for nanodomain organization

Plant cells are surrounded by a cell wall composed of a tangle of polysaccharides such as cellulose, hemicellulose and pectin. This structure enables the cell to maintain its integrity under the high internal turgor pressure. As a result, the PM is in direct contact with the cell wall. This is exemplified by protein complexes that directly link cell wall components and the PM such as CesA (Wilson *et al*., 2021). This proximity influences the diffusion of artificial PM proteins, suggesting that the cell wall may act as a corral to limit protein lateral mobility and therefore influences their distribution in the PM (Martinière *et al*., 2012). A reverse genetic screen raised for the identification of PIN polarity regulators identified an allele of *CesA3*, suggesting that PIN polarity and CesA are functionally connected (Feraru *et al*., 2011). Indeed, further investigations showed that PIN2 diffusion and cluster formation were impaired in plasmolyzed cells and after treatment with isoxaben, an inhibitor of cellulose deposition in cells (Feraru *et al*., 2011; Li *et al*., 2021). Interestingly, the lateral diffusion and the nanodomain organization of FLOT2, HIR1, FLS2, PIN3 and Plasma membrane Intrinsic Protein 2;1 (PIP2;1) proteins are affected by cell wall perturbations (Feraru *et al*., 2011; Hosy *et al*., 2015; McKenna *et al*., 2019; Daněk *et al*., 2020). Cell wall can also act on the PM organization through signaling processes involving FERONIA (FER), a prototypical isoform of *Catharanthus roseus* receptor‐like kinase gene family. FER interacts with both pectins and rapid alkalinization factor (RALF) and is acting as a cell growth regulator (Fig. 2d; Haruta *et al*., 2014; Li *et al*., 2015; Feng *et al*., 2018; Dünser *et al*., 2019; Cheung, 2024). Interestingly, upon abiotic stimulation, RALF and pectin phase separate and recruit FER together with the co‐receptor Lorelei‐Like GPI‐anchored protein 1 (LLG1) into nanodomains (Fig. 2d). This complex regulates endocytosis of noncognate receptors and therefore their downstream signaling pathways (Liu *et al*., 2024). Together, those observations show that, in plant cells, the cell wall has a crucial effect on protein organization in nanodomains. This regulatory layer can be likened to an inverted ‘picket and fence’ model.

## VIII. Functional link between PM nanodomain organization and endocytosis

The internalization of proteins from the PM is mainly achieved by clathrin‐mediated endocytosis (CME) in plant cells (Dhonukshe *et al*., 2007). Clathrin and other proteins from the CME machinery such as the Adaptor Protein‐2 (AP‐2) and the TPLATE complexes were shown to arrange in PM nanoclusters using Total Internal Reflection Fluorescence (TIRF) microscopy (Gadeyne *et al*., 2014; Johnson & Vert, 2017; Wang *et al*., 2020). Interestingly, the formation of CME nanoclusters occurs sequentially, and the TPLATE complex is recruited in PM foci, preceding the recruitment of AP‐2 complex, clathrin and dynamin‐related proteins (Gadeyne *et al*., 2014). Dual‐color variable‐angle TIRF microscopy analyses showed that clathrin (most of the time Clathrin Light Chain (CLC)) can co‐localize in nanodomains with many PM cargo proteins including the brassinosteroid receptor Brassinosteroid Insensitive 1 (BRI1), the nicotinamide adenine dinucleotide phosphate (NADPH) oxidase named Respiratory Burst Oxidase Homologue D (RBOHD), the ammonium transporter AMT1;3 and PIP2;1 aquaporin (Wang *et al*., 2013, 2015; Hao *et al*., 2014). Co‐localization between CME machinery and cargo proteins in certain nanodomains probably reflects endocytic hot spots in the PM and highlights the functional link that can exist between nanodomain organization and CME in plant cells. Endocytosis can modify the dynamics of nanodomain‐organized proteins. In response to high ammonium concentration, AMT1;3 was found to be internalized from the PM (Wang *et al*., 2013). Before internalization, the size and fluorescence intensity of AMT1;3 nanodomains increased, and concomitantly, the overall number of nanoclusters and their residence time decreased. Interestingly, CME is implicated in the dynamics of AMT1.3 nanodomains since in the *clathrin heavy chain 2* (*chc2*) mutant, the size and the fluorescence intensity of AMT1;3 nanoclusters increased (Wang *et al*., 2013). A similar enrichment in nanodomains before internalization was revealed for PIP2;1 by single‐particle tracking photoactivated localization microscopy (sptPALM) (Martinière *et al*., 2019). Indeed, a hyperosmotic treatment not only induced PIP2;1 molecule diffusion but also enhanced their local density, concomitantly to an increase in PIP2;1 internalization in root cells (Martinière *et al*., 2019).

The role of the nanodomain‐organized Flot1 protein in a putative endocytic pathway highlights the functional link between PM nanodomains and endocytosis in plants (Li *et al*., 2012). Indeed Flot1 co‐localized in PM nanodomains with numerous cargo proteins and was proposed to be involved in the internalization of BRI1, among others, since interfering with Flot1 functionality partially disturbed BRI1 endocytosis (Wang *et al*., 2015; Zhang *et al*., 2019). The Flot1‐mediated endocytic pathway probably coexists with CME and may be activated in response to environmental stimuli, as it was proposed for instance for PIP2.1 in response to salt stress (Li *et al*., 2012).

## IX. Organization of proteins in nanodomains to coordinate/modulate their functions

One of the functions of nanodomains is to gather in confined environment proteins that cooperate, and possibly physically interact, to achieve a given cellular process. In this paragraph, we will present selected examples relative to symbiosis, defense against pathogens, ion transport, hormonal and reactive oxygen species (ROS) signaling that illustrate this phenomenon.

### 1. Biotic interactions

**Fig. 3:**
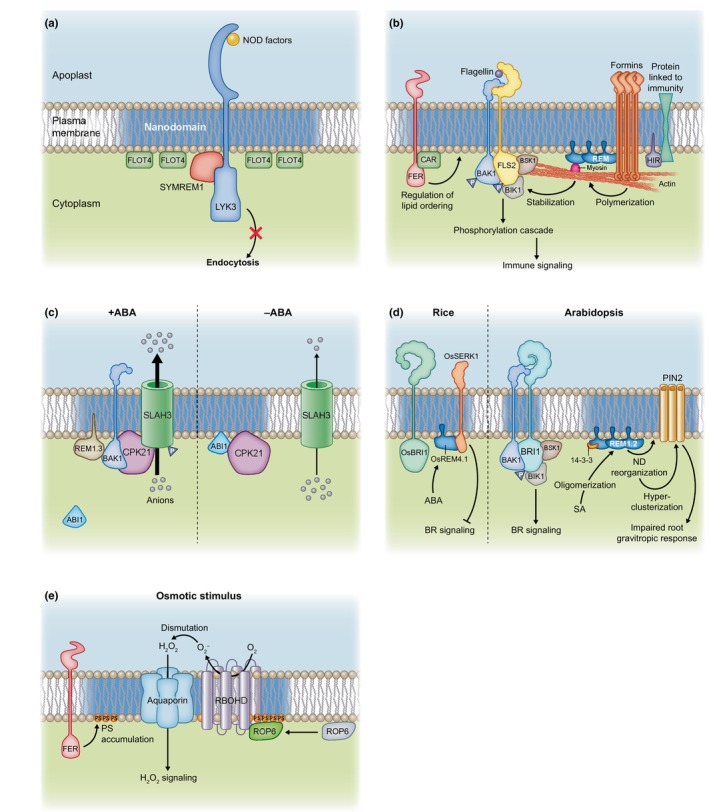
Nanodomain organization of proteins to generate regulatory/signaling hubs involved in different biological processes. (a) Symbiosis is regulated by nanodomain‐recruited protein machinery. Flotillin 4 (FLOT4)/SYMREM1 proteins act as a hub to stabilize the symbiotic receptor Lysine Motif Kianse3 (LYK3) that is involved in the perception of NOD factors. This mechanism prevents LYK3 endocytosis, thus ensuring root hair infection and the establishment of symbiosis. (b) Role of nanodomain‐organized proteins in plant immunity. In plasma membrane (PM) nanodomains, Brassinosteroid insensitive 1‐Associated receptor Kinase 1 (BAK1), Brassinosteroid Signaling Kinase 1 (BSK1) and Botrytis‐Induced Kinase 1 (BIK1) proteins interact with the flagellin receptor Flagellin‐Sensing 2 (FLS2) to initiate a phosphorylation cascade resulting in immune signaling. FLS2 nanodomains also contain Remorins (REM). FERONIA (FER) and CAR proteins physically interact and the rapid alkalinization factor 1 (RALF1)‐FER pathway induces an accumulation of CAR proteins that are recruited to the PM to stabilize the PM liquid‐ordered phase, which in turn allows the formation of the FLS2–BAK1 complex. Myosin, that allows the transport of cargoes along actin filaments, interacts with REM to recruit and stabilize BIK1 in FLS2‐containing nanodomains, which facilitates FLS2–BIK1 complex formation. Formins oligomerize and arrange in PM nanoclusters in response to flagellin, which induces their activation and further actin polymerization. This mechanism depends on the oligomerization of REM proteins that physically interact with formins. Hypersensitive Induced Reaction (HIR) are scaffolding nanodomain‐organized proteins that interact with proteins linked to immunity, although the meaning of these interactions remains to be determined. Triangles with the letter P represent phosphorylation. (c) The formation and dissociation of protein complexes in PM nanodomains regulate ion transport. The anion channel Slow Anion channel 1 Homologue 3 (SLAH3) can interact with its activating kinase CPK21 in PM nanodomains containing REM1.3, in an abscisic acid (ABA) dependent manner. In this condition, SLAH3 is fully active. In absence of ABA, the phosphatase Protein Phosphatase 2C/Abscisic acid Insensitive 1 (PP2C/ABI1) inhibits the interaction between CPK21 and SLAH3 and also probably induces a displacement of SLAH3 outside of nanodomains. This results in a reduction in SLAH3‐mediated transport. (d) Nanodomains in hormonal signaling. In rice, ABA upregulates the expression of OsREM4.1 that interacts with OsSERK1, an orthologue of Arabidopsis BAK1, to inhibit its interaction with the receptor OsBRI1. This represses OsSERK1‐catalyzed transphosphorylation of OsBRI1, thus inhibiting brassinosteroid (BR) signaling. This mechanism likely occurs in PM nanodomains. In Arabidopsis, BRI1 arranges in PM nanodomains where it interacts with BAK1, BSK1 and BIK1 to regulate BR signaling. Salicylic acid (SA) induces an accumulation of REM as well as their higher‐order oligomerization in the PM in a 14‐3‐3 protein‐dependent manner. This likely increases the liquid‐ordered phase of the PM. The SA‐mediated reorganization of nanodomains also induces a hyper‐clusterization of PIN2 at the PM resulting in an impaired root gravitropic response. (e) Reactive oxygen species (ROS) signaling in response to an osmotic signal is initiated through the cooperation of nanodomain‐organized proteins. In response to an osmotic constraint, Rho of Plant6 (ROP6) accumulates in PM nanodomains where it physically interacts with and activates Respiratory Burst Oxidase Homologue D (RBOHD) that produces superoxide ions (O_2_^−^) in the apoplast. O_2_^−^ ions are dismutated to hydrogen peroxide (H_2_O_2_) that is then likely transported across the PM by aquaporins to ensure intracellular signaling. Osmotically induced ROS production is regulated by the receptor kinase FER that stimulates phosphatidylserine (PS) PM accumulation and nanoclustering, which in turn favors ROP6 nano‐partitioning. For clarity, not all the mechanisms presented in the corresponding section of this review were illustrated in this figure.

As mentioned previously, REMs participate in PM nanodomain organization. A scaffolding function of REMs in symbiotic processes was initially highlighted by Lefebvre *et al*. (2010) who showed that SYMREM1 interacts with symbiotic receptors involved in the perception of bacterial signaling molecules and regulate bacterial infection. FLOTs were also demonstrated to be required for the establishment of symbiosis and, interestingly, the co‐localization between FLOT4 and the symbiotic receptor LYK3 in PM nanodomains from root hairs was shown to be strongly stimulated after bacterial infection (Fig. 3a; Haney & Long, 2010; Haney *et al*., 2011). Later works highlighted that both FLOT4 and SYMREM1 proteins are required to ensure symbiosis by immobilizing LYK3 in PM nanodomains (Fig. 3a; Liang *et al*., 2018). First, infection by symbiotic bacteria induces the expression of SYMREM1 that is recruited into nanodomains via a process requiring FLOT4 that probably acts as a central hub during primary nanodomain assembly. Second, SYMREM1 interacts with ligand‐activated LYK3 to stabilize the receptor into nanodomains and prevent its endocytosis, thus ensuring root hair infection.

Plant perception of pathogens involves PM‐localized immune receptors and co‐receptors that physically interact in PM nanodomains. Following binding to the bacterial flagellin, FLS2 heteromerizes with the co‐receptor Brassinosteroid insensitive 1‐Associated receptor Kinase 1 (BAK1) to initiate phosphorylation cascades resulting in immune signaling (Chinchilla *et al*., 2007; Couto & Zipfel, 2016; Stegmann *et al*., 2017). In Arabidopsis cells, FLS2 protein arranges in stable PM nanodomains that partially co‐localize with REM1.2 and REM1.3 (Fig. 3b; Bücherl *et al*., 2017; Hurst *et al*., 2023; Cui *et al*., 2024). BAK1 nanoclustering in PM was also further highlighted (Gronnier *et al*., 2022). Interestingly, the receptor‐like cytoplasmic kinases Brassinosteroid Signaling Kinase 1 (BSK1) and Botrytis‐Induced Kinase 1 (BIK1), which are involved in the transduction of the perception of flagellin via phosphorylation events, were also shown to organize in nanodomains where they physically interact with FLS2, as demonstrated by bimolecular fluorescence complementation assays (Fig. 3b; Bücherl *et al*., 2017). FER interacts with both FLS2 and BAK1 and acts as a scaffold to regulate the formation of the FLS2–BAK1 immune complex (Stegmann *et al*., 2017). Recently, FER was shown to regulate the PM nanoscale organization of FLS2 and BAK1, albeit in an opposite manner since FLS2 and BAK1 proteins were more and less mobile in *fer* mutant, respectively (Fig. 3b; Gronnier *et al*., 2022). In addition, it was proposed that FER‐mediated regulation relies on the control of lipid ordering phase in the PM via a regulation of the lipid‐binding proteins named C2 domain ABA Related (CAR) (Fig. 3b; Chen *et al*., 2023). FER and CAR proteins physically interact and the RALF1–FER pathway induces an accumulation of CAR proteins that are recruited to the PM to stabilize the PM liquid‐ordered phase. This, in turn, affects FLS2–BAK1 complex formation (Fig. 3b). Similarly to FER, leucine‐rich repeat extensins (LRX) bind RALF and are also implicated in the formation of flagellin‐induced FLS2–BAK1 complex and regulate BAK1 organization in the PM and immune signaling (Gronnier *et al*., 2022). Recently, Myosin XI, which acts as a molecular motor allowing the transport of cargoes along actin filaments, was shown to interact with REM1.3 and to recruit and stabilize BIK1 in FLS2‐containing nanodomains (Fig. 3b; Wang *et al*., 2024). This phenomenon facilitates FLS2–BIK1 complex formation and results in the activation of BIK1‐dependent defense responses upon flagellin perception. Bacterial infections are known to induce a remodeling of actin in plant cells in order to coordinate cellular processes required for plant defense (Henty‐Ridilla *et al*., 2013). As mentioned previously, this mechanism involves type‐I formins that oligomerize and arrange in PM nanoclusters in response to flagellin (Fig. 3b; Ma *et al*., 2021). This local condensation and stabilization activate formins that in turn induce actin polymerization. Recently, formin nanoclustering and actin polymerization in response to pathogens were shown to depend on REMs (Fig. 3b; Z. Ma *et al*., 2022). This mechanism relies on the capacity of REM1.2 to oligomerize through its intrinsically disordered region and to physically interact with formins. Interestingly, this signaling module can be hijacked during infection by *Xanthomonas campestris*. Indeed, the effector XopR was shown to manipulate multiple steps of actin assembly, including formin‐mediated nucleation (Sun *et al*., 2021).

The scaffolding nanodomain‐organized proteins, HIR, are involved in the defense against various pathogens in different plant species (Qi *et al*., 2011; Li *et al*., 2019; Mei *et al*., 2020). HIR proteins were shown to physically interact with proteins linked to immunity, such as (1) the immune receptor Resistant to *Pseudomonas synrigae* 2 (RPS2) that is involved in effector‐triggered immunity in Arabidopsis (Qi *et al*., 2011), (2) the rice receptor kinase named Leucine‐Rich Repeat protein 1 (OsLRR1) that has a protective role against bacterial infection (Zhou *et al*., 2009) and (3) the Pleiotropic Drug Resistance 8 (PDR8)/Penetration3 (PEN3), a camalexin exporter that confers pathogen resistance (Fig. 3b; Lv *et al*., 2017; Aryal *et al*., 2023). So far, the meaning of these interactions remains to be determined but we can hypothesize that HIR proteins are probably important to create or maintain specialized nanodomain‐localized protein hubs involved in the regulation of plant immunity. Intriguingly, proteins from pathogens were shown to interact with HIR but also with other nanodomain‐organized proteins such as REMs. Those are well known to limit the cell‐to‐cell spread of virus in plants (Raffaele *et al*., 2009; Perraki *et al*., 2018), in a phosphorylation‐dependent manner involving the CPK3 kinase (Jolivet *et al*., 2023; Legrand *et al*., 2023). Thus, the filamentous hemagglutinin‐like protein (Fha1) from *X. campestris* interacts with pepper HIR proteins (Choi *et al*., 2013). Similarly, StREM1.3 and NbREM4 interacts with the potyviral movement protein cylindrical inclusion (CI) and the *Pseudomonas* type‐III effector protein HopZ1a, respectively (Albers *et al*., 2019; Rocher *et al*., 2022). Although the biological function of the interactions between HIR/REM and pathogen effectors remains largely unknown, it probably reflects a way for pathogens to disturb the plant immunity and favor infection as illustrated by the *Turnip mosaic virus* that mediates REM1.2 degradation through the interaction with the viral protein VPg (Cheng *et al*., 2020). In the same way, the rice stripe virus (RSV)‐encoded movement protein NSvc4 can interact with NbREM1 to interfere with its S‐acylation, which affects NbREM1 PM targeting and thus prevents REM‐mediated defense against RSV (Fu *et al*., 2018).

### 2. Ion transport

Apart from symbiosis and plant immune response, the formation and dissociation of protein complexes among PM nanodomains were shown to regulate ion transport. The anion channel Slow Anion channel 1 Homologue 3 (SLAH3) can interact with its activating kinase CPK21 in PM nanodomains containing AtREM1.3 (Fig. 3c; Demir *et al*., 2013). Interestingly, this interaction is stimulated by abscisic acid (ABA) as demonstrated by Förster resonance energy transfer (FRET). Upon co‐expression with CPK21, SLAH3 abundance in DRM (considered in this study as nanodomains) increases which was interpreted by the authors as a stimulated recruitment of SLAH3 in membrane nanodomains. At the opposite, co‐expression with the Protein Phosphatase 2C/Abscisic acid Insensitive 1 (PP2C/ABI1) leads to a shift of both CPK21 and SLAH3 from DRM to detergent‐sensitive membranes (DSM, considered in this study as non‐nanodomains) (Fig. 3c). On a functional point of view, measurements of currents in Xenopus oocytes demonstrated that CPK21 activates SLAH3, which results in anion efflux whereas the co‐expression with ABI1 inhibits the interaction between CPK21 and SLAH3, thus preventing SLAH3‐mediated anion currents (Fig. 3c; Demir *et al*., 2013).

### 3. Hormonal regulations

In plants, hormonal regulation is linked to the nanodomain organization of proteins as illustrated by the brassinosteroid receptor BRI1, which clustering in the PM of Arabidopsis cells is crucial for brassinosteroid signaling (Wang *et al*., 2015; Bücherl *et al*., 2017). Interestingly, although BRI1 and FLS2 receptors employ common downstream interacting signaling components such as BAK1, BSK1 and BIK1 proteins, they organize in distinct PM nanodomains (Fig. 3d; Bücherl *et al*., 2017). Bücherl *et al*. proposed that this spatial separation of BRI1 and FLS2 in distinct domains might explain their signaling specificity in response to brassinosteroid and flagellin, respectively. An original work showed that REM can act as molecular switches to regulate the antagonistic interactions between brassinosteroid and ABA during plant development (Gui *et al*., 2016). In rice, ABA upregulates the expression of OsREM4.1 that interacts with the kinase domain of *Oryza sativa* Somatic Embryogenesis Receptor 1 (OsSERK1), an orthologue of Arabidopsis BAK1, to inhibit its interaction with the receptor OsBRI1 and repress OsSERK1‐catalyzed transphosphorylation of OsBRI1 (Fig. 3d). Interestingly, this negative regulation of brassinosteroid output can be counterbalanced by OsBRI1 itself that phosphorylates OsREM4.1 to reduce its binding affinity to OsSERK1 (Gui *et al*., 2016). In Arabidopsis, the defense hormone salicylic acid (SA) induces an accumulation of REM proteins as well as their higher‐order oligomerization and their arrangement in large clusters in the PM that are dependent on 14‐3‐3 proteins that physically interact with REM1.2 (Fig. 3d; Huang *et al*., 2019). This mechanism was proposed to be responsible for the increase in the liquid‐ordered phase of the PM observed upon SA treatment. On a functional point of view, SA‐stimulated higher‐ordered lipids, which are enriched in plasmodesmata, may decrease plasmodesmata membrane plasticity to restrict their opening and reduce virus spreading, as proposed by the authors (Huang *et al*., 2019). The SA‐mediated reorganization of nanodomains also affects auxin signaling by impairing the lateral diffusion and the endocytosis of PIN2 auxin transporter, which results in the hyper‐clusterization of PIN2 at the PM (Fig. 3d; Ke *et al*., 2021). As a result, the auxin‐dependent root gravitropic response is impaired in Arabidopsis upon SA stimulation. The nanodomain organization of PIN proteins is dependent on the PM lipid composition (Kleine‐Vehn *et al*., 2011), but may also involve protein–protein interactions. Indeed PIN1 interacts with the ATP‐binding cassette (ABC) transporter ABCB19 (Blakeslee *et al*., 2007) and *ABCB19* mutation induces a shift of PIN1 from DRM to DSM (Titapiwatanakun *et al*., 2009). However, these biochemical analyses still need to be validated by microscopy approaches. Interestingly, in a similar manner to SA, auxin was also demonstrated to modulate the PM nanodomain organization since: (1) auxin biosynthesis mutants exhibit reduced lipid ordering in the PM of Arabidopsis pavement cells and (2) auxin induces the nanoclustering of the auxin‐related receptor kinase TMK1 and increases the size of FLOT1‐containing nanodomains (Pan *et al*., 2020).

### 4. Reactive oxygen species

Reactive oxygen species signaling in plant cells is initiated through the cooperation of nanodomain‐organized proteins and is regulated by environmental factors. A key player is the PM‐localized NADPH oxidase named RBOHD that clusters in nanodomains. Upon activation, RBOHD generates superoxide in the apoplast, which turns to H_2_O_2_ by dismutation (Hao *et al*., 2014). Then, H_2_O_2_ is likely transported across the PM by channels such as PIP aquaporins (Bienert *et al*., 2007; Bienert & Chaumont, 2014; Rodrigues *et al*., 2017). In response to an osmotic signal, ROP6 is accumulating in PM nanodomains where it physically interacts with and activates RBOHD to produce ROS that will act as secondary messengers, inducing an adaptive response of the plant to the osmotic constraint (Fig. 3e; Smokvarska *et al*., 2020). Interestingly, although auxin also induces ROP6 nanoclustering (Platre *et al*., 2019), the corresponding nanodomains do not contain RBOHD and differ from osmotically induced ROP6 nanodomains. This suggests that ROP6 nano‐partitioning at the PM ensures signal specificity downstream of independent stimuli (Fig. 3e; Smokvarska *et al*., 2020). As recently demonstrated, osmotically induced ROS production is regulated by the receptor kinase FER. It controls ROP6 nanoclustering through the modulation of PS nanodomain density (Fig. 3e; Platre *et al*., 2019; Smokvarska *et al*., 2023). Thus, FER signaling regulates the strength of ROP6 signaling by controlling the density of ROP6 nanodomains (Smokvarska *et al*., 2023). The production of ROS in response to pathogens is essential for plant immunity (Kadota *et al*., 2014). Upon flagellin perception, BIK1 directly interacts with and phosphorylates RBOHD to stimulate its activity (Kadota *et al*., 2014; Li *et al*., 2014). However, although both proteins were independently shown to arrange in PM nanodomains (Hao *et al*., 2014; Bücherl *et al*., 2017; Smokvarska *et al*., 2020), the interplay between their physical association and their nanodomain organization remains to be determined.

## X. From nanodomains to nanoenvironments

**Fig. 4:**
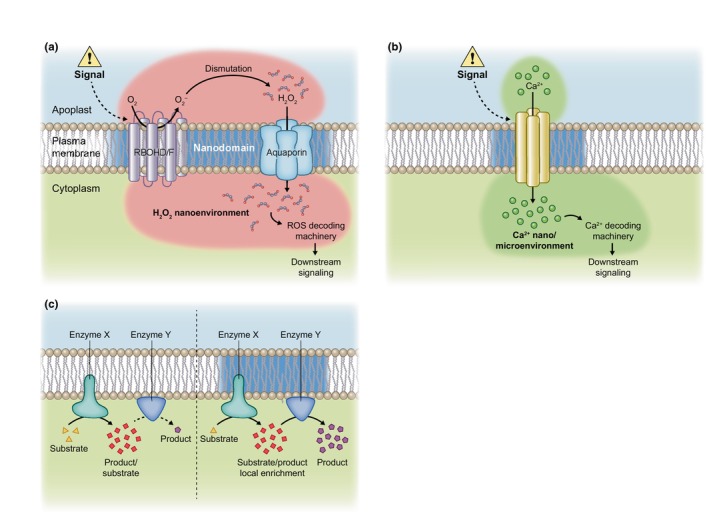
Nanoenvironments to generate signaling pathways. (a) Model for signal‐induced reactive oxygen species (ROS) nanoenvironment at the plasma membrane (PM). Upon stimulus, nicotinamide adenine dinucleotide phosphate oxidases RBHOD and F are activated leading to the production of superoxide ions (O_2_^−^) in the apoplast that are then dismutated to H_2_O_2_, which in turn is likely transported inside the cell by aquaporins. We propose that this mechanism leads to a highly localized oxidation in both the apoplast and the cytoplasm at the immediate vicinity of the PM that we call H_2_O_2_ nanoenvironment. This H_2_O_2_ accumulation in the cytoplasm may cause the oxidation of molecular actors of the ROS decoding machinery that would be, for some of them, organized in clusters at the cytosolic face of the PM. This activates the downstream signaling. (b) Model for signal‐induced calcium nano/microenvironment at the PM. Following the same principle than for H_2_O_2_ nanoenvironment, upon stimulus, calcium channels putatively present in PM nanodomains are activated leading to a localized accumulation of calcium ion in the cytosol close to nanodomains. Molecular actors of the calcium decoding machinery that may be, for some of them, organized in PM clusters would then be activated. (c) Model for nanometric organization of enzymatic complexes to generate channeling processes. Efficiency of enzymatic reaction relies on biochemical environment and substrate availability. Here, we propose that clustered enzymatic complexes could be a way to locally enrich substrates, especially in the case of multi‐step reactions involving several enzymes, to optimize the production of certain molecules. We can imagine that the relocalization of certain enzymes among enzymatic clusters upon signals could be a way to activate the pathway.

Molecular processes are compartmentalized inside the cell for proper functioning. The nanoscale organization of the PM brings the importance of this compartmentalization at further scale. Indeed, the co‐clustering of biomolecules involved in signaling, as described upon osmotic signal with the ROP6 GTPase and the ROS producing enzymes RBOHD/F (Smokvarska *et al*., 2020), raises questions about the role of the localized production of secondary messengers. An appealing hypothesis is that this localized ROS production leads to the formation of ROS nanoenvironments at the immediate vicinity of the PM that is mandatory for the establishment of molecular responses (Fig. 4a). Such nanometric RBOH‐dependent ROS production/accumulation involved in signaling was documented in tobacco leaves (Lherminier *et al*., 2009). Using CeCl_3_ staining under transmission electron microscopy, the authors described nanometric ROS accumulation at the immediate vicinity of the PM, on the cytosolic face, in response to the cryptogein, a fungal elicitor. Those cerium deposits of 100 nm in diameter reflecting ROS accumulation appear within minutes in elicited cells in a RBOH‐dependent manner (Lherminier *et al*., 2009). In the context of lateral root emergence, a similar pattern of CeCl_3_ precipitates was shown at the contact between endoderm and the lateral root primordia. In this case, RBOH activity was suggested to help lateral root emergence (Orman‐Ligeza *et al*., 2016). A local H_2_O_2_ accumulation was also demonstrated during the differentiation of endodermal cells and the Casparian strip formation (Lee *et al*., 2013; Fujita *et al*., 2020). Whereas a lateral sequestration of ROS in the plane of the PM is clear from localization of sub‐micrometric CeCl_3_ precipitates, it remains undetermined whether H_2_O_2_ nanoenvironments exist on both sides of the PM. This is likely the case since superoxide produced by RBOH and the resulting H_2_O_2_ accumulate in the apoplast, but then H_2_O_2_ is probably transported across the PM by PIP aquaporins, as mentioned previously, thus generating H_2_O_2_ hot spots on the cytoplasmic side. Recently, Kritsiligkou *et al*. (2023) showed interesting insights in yeast that illustrate the existence of H_2_O_2_ nanoenvironments (Kritsiligkou *et al*., 2023). They fused the H_2_O_2_ sensor, HyPer7 to the C‐terminus of all different open reading frames of the yeast genome. This approach led to the identification of differentially oxidized individual proteins and protein complexes and illustrated a compartmentalization of the ROS signaling that was more sophisticated than previously admitted.

A local enrichment of secondary messengers is not exclusive to ROS. Localized accumulation of calcium ions forming ‘calcium hot spots’ ranging from a few nanometer scale to several micrometers have been first documented in neuronal cells (Augustine *et al*., 1992). Generated aside calcium channels at the inner side of the PM, they are usually referred to as calcium microdomains (Fig. 4b). Interestingly, calcium release at the PM activates Syntaxin 1, a SNARE protein that regulates exocytosis. Calcium acts as a charge bridge that specifically and reversibly connects multiple Syntaxin 1 and PIP(4;5)P (Milovanovic *et al*., 2015, 2016). Consequently, calcium microdomains may drive vesicle fusions in specific zones of the PM. Studies of certain cation channels like Two‐Pore Channel 1 (TPC1) showed that their full activation would need high cytosolic Ca^2+^ concentration that would be detrimental for cell survival in case of global calcium release (Pottosin & Dobrovinskaya, 2018). However, a local accumulation of calcium among a nano/microdomain would circumvent this problem by allowing both the activation of the channel and cell survival. Most evidences concerning local accumulation of secondary messengers creating nanoenvironments are indirect in plants. Undoubtedly, the recent technical advances in super‐resolution microscopy combined with new genetically encoded sensors for secondary messengers like HyPer7 for H_2_O_2_ (Ugalde *et al*., 2021) and GCaMP‐based sensors for calcium (Waadt *et al*., 2017), will allow in the future to formally demonstrate the existence of these nanoenvironments.

Signaling pathways emerging from nanoenvironments obligatory require a localized decoding machinery (Fig. 4a,b). The ROS signaling decoding machinery would rely on sensing proteins, containing cysteines highly sensitive to H_2_O_2_, that transmit the primary oxidation to protein thiols and so on to downstream target proteins (Meyer *et al*., 2021; Mittler *et al*., 2022). This requires strong proximity of the involved molecular actors and protein–protein interactions. Interestingly, some glutathione peroxidase (GPX), thioredoxin (TRX) or peroxiredoxin (PRX) could be membrane associated and would typically participate in oxidation of target biomolecules (Delaunay *et al*., 2002; Attacha *et al*., 2017; Bi *et al*., 2022). Interestingly, the LRR receptor kinase H_2_O_2_‐induced CA2+ increases 1 (HPCA1) is activated by H_2_O_2_ application through the covalent modification of its extracellular cysteine residues leading to its autophosphorylation (Wu *et al*., 2020). This demonstrates that H_2_O_2_ decoding could happen at the PM and may rise from apoplastic H_2_O_2_ nanoenvironments. The calcineurin B‐like (CBL) proteins are important actors of the calcium decoding machinery in plants. Together with CBL‐interacting protein kinases (CIPKs), they form protein complexes that phosphorylate target proteins mediating molecular responses. CBL proteins are localized to the PM through lipid modifications (myristoylation and S‐acylation) and are major candidates for calcium decoding proteins among nano/microdomains (Batistič *et al*., 2008). CPK proteins are other molecular actors of the calcium decoding machinery in plants. Interestingly, CPK3 activation by virus induces a decrease of its diffusion in the PM and leads to its confinement in nanodomains. A similar behavior is observed using a constitutively active form of CPK3, linking the kinase activation to its PM organization (Jolivet *et al*., 2023). The study of such precise organization of signaling decoding complexes, for example CBL/CPK for Ca^2+^ signaling or GPX/TRX/PRX for ROS signaling is a promising field of research that gives new insights in the spatiotemporal molecular responses of plant cells. Given the numerous cross talks between ROS and calcium signaling, it is likely that ROS and calcium nanoenvironments could overlap and regulate each other depending on situations.

In a more general way, nanometric accumulation of molecules may participate in the metabolite channeling of enzymatic activities, also called metabolons. Metabolite channeling processes consist in the transfer of the product of a proximal activity directly to a distal activity as a substrate, without equilibration with the bulk solvent, thereby enhancing the efficiency of the kinetic process (Fig. 4c; Kuzmak *et al*., 2019; Pareek *et al*., 2021; Dahmani *et al*., 2023). At this stage, the exact role of membrane nanodomains in metabolite channeling remains poorly explored in plants.

## XI. Conclusion and future prospective

Our view of biological membranes has constantly evolved since the lipid bilayer model proposed in the 1920s by Gorter & Grendel (1925) and the fluid mosaic model by Singer & Nicolson (1972). Nowadays, the membrane's lateral heterogeneity in lipids and proteins forming nanodomains is well accepted.

As fluorescent microscopy techniques advance toward higher resolution, the study of plant nanodomains has emerged as a key area in plant cell biology, helped by the development of genetically encoded biosensors. Because nanodomains induce high local concentration and/or low diffusion of lipids and proteins, they are predicted to shape chemical reactions and would bring new avenues to understand cellular processes.

In the plant biology field, numerous studies have demonstrated that plant membrane proteins can be organized in nanodomains (Ott, 2017; Gronnier *et al*., 2018; Jaillais & Ott, 2020; Martinière & Zelazny, 2021; Jaillais *et al*., 2024). Nevertheless, our understanding of how membrane nanodomain organization participates in cellular function has just started to emerge. Nanodomains have a scaffolding function that mediates protein–protein interactions necessary for diverse processes such as symbiosis, immune signaling or ion transport. This scaffolding role can participate in the regulation of protein post‐translational modification, for example phosphorylation (Demir *et al*., 2013; Chu *et al*., 2021). Thus, specific nanodomains may act in segregating kinases or phosphatases together with their target proteins. Nanodomain organization of membranes may participate in signal specificity. Even if they share common downstream signaling components, FLS2 and BRI1 form distinct nanodomains in the PM. This could explain why they maintain distinct signaling output in immunity and steroid‐mediated growth, respectively (Bücherl *et al*., 2017). Importantly, nanodomains should be seen as highly dynamic structures whose organization is modified by environmental stimuli as exemplified by ROP6 protein that organizes into nanodomains to ensure ROS signaling in response to osmotic or auxin stimulations (Platre *et al*., 2019; Pan *et al*., 2020; Smokvarska *et al*., 2020).

Nanodomain organization and maintenance are complex and require specific lipids, oligomerization of ‘driver’ proteins, the cytoskeleton and the cell wall. One of the main objectives in the future will be to understand how these different parameters are orchestrated and interconnected. Concerning the interplay between lipids and proteins, an interesting aspect to explore is the capacity of some ‘driver’ proteins, such as REMs, to directly interact with lipids to potentially shape nanodomains (Perraki *et al*., 2012; Legrand *et al*., 2023). Another very interesting aspect would be to determine to what extent microtubules and the cell wall, which are in constant dialog during cell growth, cooperate to organize nanodomains. Along this line, recent studies showed a functional interplay between stress sensing, CesA complex localization/dynamics and microtubule organization (McFarlane *et al*., 2021; Kesten *et al*., 2022).

Secondary messengers such as Ca^2+^ and ROS participate in a myriad of signaling processes. They are at the neck of hourglass signaling pathways, being induced by various stimuli and leading to specific downstream cellular responses. Even if it remains rather speculative at this stage, in this review we propose that plant nanodomains may participate in the formation of local chemical nanoenvironments, in the absence of any diffusion barriers like membranes. Such heterogeneity in Ca^2+^ and H_2_O_2_ concentrations or difference in the pH could be later decoded locally by cells. This hypothesis is supported by indirect evidence: the nanodomain organization of proteins involved in secondary messenger production, for example RBOHs (Hao *et al*., 2014; Smokvarska *et al*., 2020); a local enrichment of certain secondary messengers close to the PM (Lherminier *et al*., 2009; Orman‐Ligeza *et al*., 2016; Fujita *et al*., 2020); and PM‐localized protein involved in secondary messenger decoding, for example GPX/TRX, CBLs/CPKs (Batistič *et al*., 2008; Attacha *et al*., 2017; Jolivet *et al*., 2023). According to this model, and because of their proximity in the same type of nanodomains, the activation of a given secondary messenger producer may locally induce the appropriate decoding machinery, and would thus contribute to the generation of a specific signaling signature.

## Competing interests

None declared.

## Disclaimer

The New Phytologist Foundation remains neutral with regard to jurisdictional claims in maps and in any institutional affiliations.
